# Conversations about Death and Dying with Older People: An Ethnographic Study in Nursing Homes

**DOI:** 10.3390/healthcare6020063

**Published:** 2018-06-14

**Authors:** Åsa Alftberg, Gerd Ahlström, Per Nilsen, Lina Behm, Anna Sandgren, Eva Benzein, Birgitta Wallerstedt, Birgit H. Rasmussen

**Affiliations:** 1 Department of Social Work, Faculty of Health and Society, Malmö University, SE-205 06 Malmö, Sweden; Asa.Alftberg@mau.se; 2 Department of Health Sciences, Faculty of Medicine, Lund University, P.O. Box 157, SE-221 00 Lund, Sweden; Lina.Behm@med.lu.se (L.B.); Birgit.Rasmussen@med.lu.se (B.H.R.); 3 Department of Medical and Health Sciences, Division of Community Medicine, Linköping University, SE-581 83 Linköping, Sweden; Per.Nilsen@liu.se; 4 Center for Collaborative Palliative Care, Department of Health and Caring Sciences, Faculty of Health and Life Sciences, Linnaeus University, SE-351 95 Växjö, Sweden; Anna.Sandgren@lnu.se (A.S.); Eva.Benzein@lnu.se (E.B.); Birgitta.Wallerstedt@lnu.se (B.W.); 5 The Institute for Palliative Care, Region Skane and Lund University, P.O. Box 157, SE-221 00 Lund, Sweden

**Keywords:** auxiliary nurse, existential communication, frailty, ethnographic approach, life-limiting disease, older, aged, palliative care, residential care, end-of-life

## Abstract

Nursing homes are often places where older persons “come to die.” Despite this, death and dying are seldom articulated or talked about. The aim of this study was to explore assistant nurses’ experiences of conversations about death and dying with nursing home residents. This study is part of an implementation project through a knowledge-based educational intervention based on palliative care principles. An ethnographic study design was applied in seven nursing homes, where eight assistant nurses were interviewed and followed in their daily assignments through participant observations. The assistant nurses stated that they had the knowledge and tools to conduct such conversations, even though they lacked the time and felt that emotional strain could be a hinder for conversations about death and dying. The assistant nurses used the strategies of distracting, comforting, and disregarding either when they perceived that residents’ reflections on death and dying were part of their illness and disease or when there was a lack of alignment between the residents’ contemplations and the concept of dying well. They indicated that ambivalence and ambiguity toward conversations about death and dying should be taken into consideration in future implementations of knowledge-based palliative care that take place in nursing homes after this project is finalized.

## 1. Introduction

Palliative care encompasses conversations that are expected to help dying individuals prepare for death, to create a closing of what has been (life) and an opening to what is to come (death). This means that dying individuals should receive the opportunity to express their wishes, needs, and feelings, and obtain insight into their own dying [1,2,3,4]. Being able to express thoughts and feelings about death and dying is regarded as fundamental to quality of life, provided that the dying person wants to talk about these issues [5], and this can be described as a spiritual need that should be addressed by medical staff [6]. Accordingly, society’s expectations of palliative care staff are that they are to facilitate such expressions and insights by encouraging nursing home residents to talk about their feelings and thoughts about death and dying. Such conversations are expected to promote a good and dignified death, which also can be regarded as a cultural concept or ideal [7].

The World Health Organization (WHO) has called for improved palliative care for older persons in general [4]. Older people dying of multiple morbidities, or merely of “old age,” have until recently received far less of this type of care [8]. In several countries around the world, a high proportion of all deaths among older people over 65 years occur in residential care facilities [9]. This is the case in Sweden, where 24.5% of all deaths among those aged 70 years and older occur in nursing homes; this figure increases to 62% for people aged 90 years and older [9,10]. The Swedish policy is to support older people to live normal lives in their own homes for as long as possible [11]. This has contributed to the situation whereby the oldest and frailest persons move to nursing homes, with an average life expectancy in Swedish nursing homes of six to nine months; this time frame has reduced in recent years [12]. In addition, relatively few individuals living in nursing homes are transferred to hospitals to die there [10]. This means that the staff in nursing homes are given the role of handling death and dying and to provide “a good dying process” [1,13]. 

The principles of palliative care that have been developed within specialized palliative care and hospices may be lacking in nursing homes, since they traditionally have not been part of regular education or training [4,14,15] of staff in specialized palliative and oncology units. While staff in nursing homes strive to provide good care for older persons in their final stages of life, practical routines and everyday life rather than death and dying are considered more appropriate topics to discuss with the residents. Emotional and existential needs and concerns tend to be avoided due to uncertainty about how to talk about such topics [16,17,18]. Matters that relate to death and dying have been found to create emotional strain and even evoke fear among the staff, who are uncertain about how to face a dying person’s suffering [19,20]. Previous studies have shown a lack of communication about existential issues such as death and dying between staff and older persons [21,22,23,24]. To remedy this situation, several attempts to implement palliative care in nursing homes are described in the literature, for example through education [8,17,25] and Advance Care Planning (ACP) [26,27,28]. 

In a recent publication on mapping palliative care implementation activities on the macro-, meso-, and micro levels in 29 European countries, Froggatt et al. [8] identified low levels of palliative care development and delivery in nursing homes, although there is great variation and diversity among countries. Palliative care is an integrated part of the Swedish health care system, including national guidelines for good palliative care at the end of life [29] and a national program for palliative care [30]. However, the quality of palliative care is still substandard and random for many residents living in nursing homes [12,14,31].

The Swedish system of health care and social services is financed primarily by taxes. The provision of long-term care and services for older people is within the remit of municipalities and is preceded by needs assessments. The most common staff members employed in Swedish nursing homes are assistant nurses (ANs). They generally have a shorter education (secondary school and 6–12 months of practical training for working in elder care and in the hospital) than registered nurses. They work most closely with the nursing home residents, while supervised by a few registered nurses. Many tasks are formally delegated by registered nurses to ANs, who then provide everyday care, including such conversations with the residents as studied here. At the national level, the ratio of ANs to RNs is 25:1 [32].

In agreement with ongoing efforts to improve palliative care in nursing homes, an implementation project called Implementation of Knowledge-Based Palliative Care in Nursing Homes (Swedish acronym KUPA) was performed in 2015–2017. The educational intervention was provided to nursing home staff and managers, addressing knowledge and skills of relevance to develop evidence-based palliative care [33]. The importance of context has increasingly been emphasized in implementation research [34], and conversation can be seen as one key aspect of context. This study explores conversation in the context of the ongoing implementation of palliative care in nursing homes based on education to support staff. This knowledge may contribute to increasing understanding of the outcomes of the implementation process and context in future implementation studies. 

## 2. Aim

The aim of this paper is to explore assistant nurses’ experiences of conversations about death and dying with nursing home residents within the framework of an ongoing implementation of palliative care. 

## 3. Materials and Methods 

This study is based on an ethnographic design with fieldwork. By applying an ethnographic method using both participant observations and interviews, a deeper understanding of concrete conversations about death and dying in the nursing home setting can be achieved. Following assistant nurses (ANs) in their everyday work made it possible to study their experiences in context. By the term “conversations about death and dying,” we refer to situations where the residents bring up issues that somehow concern their death and how the ANs respond.

### 3.1. Context of the Study

The fieldwork was performed as part of the KUPA implementation project concerning palliative care for older people in nursing homes [33]. The project consists of a complex intervention through educational seminars that address the knowledge and skills required to achieve knowledge-based palliative care in nursing homes. The seminars involved 20 nursing homes over 6 months. The seminar covered the importance of talking about dying and death and the participants’ own feelings and thoughts about these issues, and highlighted the significance of palliative care, support for next of kin, symptom relief, and collaborative care. The intervention was evaluated before, during, and after implementation through an experimental crossover design with intervention and control groups. The KUPA has the clinical trial registration number NCT02708498 [33]. 

This study was carried out in seven nursing homes in southern Sweden. The nursing homes were strategically selected from the 20 nursing homes included in the KUPA project, reflecting diversity in size and location, a mix of larger and smaller nursing homes, and between towns and the countryside. Three nursing homes were located in rural areas: with 35, 54, and 78 beds, respectively, and four nursing homes in urban areas with 26, 48, and 90 beds, respectively. The mean nursing staff to resident ratio was 0.9:1.

### 3.2. Participants

Eight ANs participating in ongoing seminars of the implementation project in the seven nursing homes were included. Their attention to issues of death and dying was of interest to inform the implementation process in the KUPA project, which motivated the timing of this study.

They were selected after contact with the nursing home managers. The managers forwarded a request to the ANs in the educational seminars, who then voluntarily decided to be part of the ethnographic study. In one nursing home, two ANs chose to participate. They were female, 30 to 64 years old (median 57 years), and had been working as ANs in elderly care for between 10 and 30 years (median 22 years).

### 3.3. Data Collection in the Ethnographic Fieldwork

The intention was to follow each of the eight ANs on four occasions (two ANs were followed on two occasions) in their daily work through participant observations by the first author on 28 occasions in total. Each period of observation lasted 3 to 4 h (about 110 h total). The observations were made at different times of day, from 7:00 a.m. to 9:00 p.m. The first author shadowed the participating ANs in every part of their work.

During the observations, informal conversations occurred between the ANs and the first author, which later were recorded as field notes together with descriptions of the situations that took place at the nursing homes [35].

On the last occasion of observation in each nursing home, semi-structured interviews with the participating ANs were performed, containing questions about their views and experiences of existential issues such as death and dying. Questions were based on the observations and the specific situations that had occurred (“How did you feel when…?” “What did you think when…?”). The eight interviews lasted around 45 min on average, were recorded digitally, and were transcribed verbatim as well as validated by the first author. 

### 3.4. Data Analysis

A qualitative inductive thematic analysis process was applied [36] by the first, second, and last author. Field notes from the observations (which included informal conversations between the first author and ANs) and the semi-structured interviews were read as a whole to get an overall understanding of experiences and communication in the context of death and dying. Next, meaning units, i.e., text segments that related to death and dying, were identified and coded for their content, and were then sorted and grouped into clusters. Then the codes and meaning units in each cluster were reread and compared and searched for similarities and differences, forming and supporting the development of two basic themes: (1) barriers to conversations about death and dying and (2) managing conversations about death and dying in practice, i.e., in encounters between ANs and residents. The analysis continued by rereading and reflecting on the codes and meaning units, and this meant examining and recording patterns within and between the two themes, leading to the construction of six subthemes. In the group of co-authors, the interpretations made during the analysis featured a reflexive consideration, where understandings and interpretations were critically examined and discussed. 

## 4. Ethical Considerations

The study was guided by the research ethical principles for medical research (the Declaration of Helsinki) and approved by the Regional Ethics Review Board in Lund (reference number: 2015/4). 

During the fieldwork, the interactions between ANs and nursing home residents were observed, and all were informed and gave consent to be part of the research project. The residents were regarded as a vulnerable group, and the presence of the first author was guided by responsiveness towards their reactions to the observations. Any hesitation or unwillingness to participate was respected, and in some delicate situations (for instance, helping residents with intimate care or dealing with residents with severe dementia), the observations were stopped at the first author’s own initiative. No personal data about staff or residents was recorded during the observations. In the account of the results, characteristics of the ANs and the residents have been changed or omitted, and they have been given assumed and neutral names to protect their identities.

## 5. Results

The first theme, barriers to conversations about death and dying, contains two subthemes: lacking time and feeling emotional strain. The second theme, managing conversations in practice, contains four subthemes: having tools, distracting, comforting, and disregarding. The ethnographic method designates that the subthemes are presented with extensive excerpts, which are presented as cases below, as being representative of the total dataset. 

### 5.1. Barriers to Conversations about Death and Dying

All the ANs highlight the importance of being prepared to talk about death and dying whenever residents express their thoughts on the subject. Nevertheless, they perceive two barriers in particular, as described below. When the ANs talk about the experience of having conversations about death and dying with the residents, they describe feeling that lack of time is a difficulty. The ANs can also experience emotional strain in relation to these conversations.

#### 5.1.1. Lacking Time

The context aspect of time is raised continuously by the ANs. They sometimes have very little time to actually pick up the thread and continue a conversation when a resident unexpectedly wants to talk about death and dying. Melissa describes how she tries to manage this:

It may be that I don’t have time to sit down and talk about it, sometimes the timing is bad. But you have to try, if you can’t talk at that moment you’ll have to ask, nicely, if you can come back later when things are not so busy when I really feel that I have a moment to spare, and then we can talk.

However, some of the ANs claim that one should not postpone such a conversation. Even if time is lacking, due to staff shortage, for example, one has to seize the opportunity. Elizabeth says:

It must be when it happens, that’s when I have to take the time to… you try to, you’ll have to sacrifice, well sacrifice is perhaps not the right word, but I have to take time to listen if they want to talk. Sometimes you feel you don’t have the time, really. That’s awful. But you try as much as you can. And everyone at work thinks the same, and we understand if someone takes their time with one of the residents, it’s like “well, she’s still in there, probably something is going on” and you won’t disturb.

The ANs experience that lack of time may interfere with the residents’ desire to talk about death and dying, and they try to manage time constraints. 

#### 5.1.2. Feeling Emotional Strain

Apart from lack of time, another barrier to conversations about death and dying is that it may cause emotional stress that can be exhausting for the ANs. Rebecca expresses that it can be difficult to come as close to another person as one does when talking about death and dying, and she occasionally wishes she did not have to: “It may sound weird I guess, but to not have to listen. Not having to know everything about that other person.” Working as an AN and caring for older persons’ bodies (washing, dressing, and feeding) is different than caring for existential needs, which, according to Rebecca, is much more strenuous. She describes that taking part in the residents’ reflections on death and dying feels almost too intrusive. Sarah remarks on the intimate nature of care work from another perspective. She refers to how staff and residents are “feeling as one big family,” but that also makes the work exhausting:

It can drain your energy, sometimes you don’t have the strength to sit and talk. … We try to give them [the residents] so much energy, and we try to take away their negative emotions. But sometimes it’s too much.

To sum up, conversations about death and dying appear to involve emotional strain for the ANs, which may act as a barrier to carrying out these conversations. 

### 5.2. Managing Conversations in Practice

This section illustrates different strategies that ANs apply when it comes to managing conversations about death and dying with residents. The ANs point out that they have received knowledge and tools to conduct conversations about death and dying, and they give examples of strategies that they find helpful to use. However, most of the examples that were observed during the fieldwork included strategies used to circumvent conversations about death and dying. Distracting contains aspects of comfort, but it is mainly about redirecting a resident’s attention to something other than death and dying. Comforting illustrates how the ANs truly focus on the residents, even though the comfort in itself may connote distraction, while disregarding is an evasive strategy, avoiding the perceived potential risk of talking about death and dying. 

#### 5.2.1. Having Tools

The ANs say that they appreciate having the opportunity to receive more knowledge by participating in the seminars of the implementation project. Sharing experiences and knowledge with colleagues working in a similar context is highly valued. They describe experiences of having acquired tools, including knowledge of how to talk about dying and death, through the seminars. Barbara gives an example of how one dying resident returned from the hospital, immediately saying, “I’m just coming home to die.” Barbara says that she was a little bit surprised by this statement, but as she had recently learned to do, she asked the woman what thoughts she had about her dying. The resident replied that it felt “bright” and she was not afraid. Barbara emphasizes that it was a short conversation, but it felt nice to have support and knowledge from the seminars and tools such as asking questions about the resident’s thoughts when she brought up the topic. She senses that she has learned to talk about death and dying in a way that she could not before. It is very much a question of listening and confirming, she explains. “One mustn’t be afraid of these issues,” she says. “Perhaps we are not so good at talking about it as we should be, but I do feel better if the residents are comfortable with their coming death.”

The ANs express a wish to improve how to talk about death, which the seminars have promoted. Many of them state that death and dying usually is a topic that they mainly come in contact with when residents begin to express that they no longer have the strength to carry on. Melissa refers to a typical situation in her work:

I think these conversations start when you have arrived at final phase, when they are really ill, not before. It happens all of a sudden; one of the residents was washing at the washbasin and suddenly she asked me: “Am I going to die here?” At first, I was surprised and didn’t know what to say. I think I answered that she could stay here as long as she wanted and that maybe she was going to die here, nobody can tell. Such things can come all of a sudden. Sometimes you reply, “Oh no, don’t think like that, you’re healthy right now,” and you try to encourage them, but I have learned now that this is wrong, you should ask another question instead: “What are you thinking now?” rather than saying “You shouldn’t think about that, you’re not there yet, you’re healthy right now.

The ANs demonstrate an awareness of what ought to be the right response to the older person’s questions and reflections. They highlight that the residents’ reflections emerge in the very final stage of life, often at unexpected moments, and commonly concern more practical matters about the funeral. Overall, when the residents talk about death and dying, the ANs feel they have received more knowledge and a strategy, which is to listen and to ask questions. 

#### 5.2.2. Distracting

Even if the ANs feel more skilled and secure, there are still situations where they do not use the strategy of listening and asking questions, but rather a strategy of distraction. The following example is representative of this strategy. Mildred, a resident, is lying in bed when the AN, Rebecca, knocks on her door and enters her room. Rebecca asks Mildred if she wants some supper, but Mildred declines. Mildred says there is no use eating. “I’m not going to lie here much longer, I hope,” she says. Rebecca gives a soft laugh and says “all right” while putting a blanket over Mildred in her bed. Mildred then says that she wishes she had died when she had a fall and injured herself badly, something that happened several years ago. ”But think of all you would have missed. All your great-grandchildren,” Rebecca replies. “Yes,” Mildred says, and tells a story of one of her great-grandchildren. 

Rebecca later explains that Mildred talks a great deal about death. According to Rebecca, Mildred is ready to die, but at the same time she is afraid. She is in pain and wants more medication, but Rebecca believes that she may suffer from spiritual angst rather than physical pain, with the medications intended to reduce her anxiety. Mildred’s greatest fear is to be buried alive before being completely dead, and Rebecca has promised to make sure that Mildred is dead by sticking her finger with a needle or knife when that time comes. They have also talked about other aspects of dying. For example, Rebecca has explained what will happen to Mildred after she dies: the staff will wash her gently, dress her in fine clothes, and make her bed and room look nice and tidy. Sometimes Mildred says that she will try to kill herself, using a razor blade or a pair of scissors. Rebecca finds it difficult to know how to respond at such moments.

Well, sometimes you don’t give a very wise answer, I guess. It’s more like “Think of your children, do you really want them to remember you like that?” and then she goes “Well, no, I don’t.”

Hence, dying and death *is* a recurring topic of conversation between Mildred and Rebecca. Perhaps that is why Rebecca, in the situation described above, does not try to elaborate, but instead attempts to distract Mildred by talking about her great-grandchildren. Mildred may be perceived as dwelling upon death without getting closer to being prepared for it with a peaceful mind, and distraction is found to be the best solution to manage this.

#### 5.2.3. Comforting

Close to the distracting strategy is the strategy of comforting. The ANs are expected to give comfort to the residents and improve their well-being in whatever way they can. It is a natural part of their work, which is illustrated in the following strategy.

Alice, a resident known to have severe anxiety and suffer from panic attacks, is seated by her kitchen table at the window. She looks sad and worried. “I’m sitting here, waiting for the end,” she says. She explains that she has trouble breathing, and she is taking short, quick breaths. Barbara sits down calmly beside her and asks in a gentle tone if Alice wants some water. Alice does not answer, but continues to say that she is near the end now. Barbara says “Oh, no” with a comforting voice and squeezes Alice’s hand. She reminds Alice that she has had this feeling several times before, and it will pass. Barbara soothingly pats Alice’s back and tries to ease her by talking about the coming musical entertainment this afternoon and says that Alice may be feeling better by then. Alice says that she would feel better if she could get some medication. Barbara takes her request seriously and unlocks the medication locker in the kitchen and checks the medication signing list. Alice received a mild sedative earlier in the morning, and Barbara asks Alice if she could take aspirin instead. Alice says she prefers something stronger. Barbara notes that she has to phone the nurse in that case, and she takes her phone from her pocket. Talking to the nurse, Barbara explains that she wants to give Alice an aspirin, and the nurse asks if Alice is in pain. “No, she’s worried and she’s not feeling well,” Barbara says. The nurse and Barbara agree to give Alice two aspirin, saving the second daily dose of the sedative for the evening. Alice seems to be more relaxed now and content with the solution, and she takes the two aspirin Barbara gives her. Barbara gives Alice an encouraging smile and tells her she will be back later, in time for lunch, to check how she is feeling.

Alice’s anxiety has consequences for the way she is treated. When Alice says that she is sitting there and waiting for the end, it is not regarded as an opening to a conversation about death. Instead, it is interpreted as an illness that needs to be treated. Barbara manages the situation by listening to Alice and trying to comfort her, but she does not ask any questions about Alice´s thoughts about death and dying. 

#### 5.2.4. Disregarding

Disregarding is a strategy to avoid listening or talking about death and dying. This may occur when the AN perceive risks with conversations about death and dying, as in the next example. Jean, a resident who suffers from mild dementia, is sitting in the corridor on her walker. When I (the first author) walk by, she turns to me and says that there is no point anymore. “I feel so alone,” she tells me, and looks sad. “I don’t know what to do,” she says. One of the ANs, Pamela, arrives, and she seems to be in a hurry. She does not answer Jean, but suggests that Jean could water the flower pots in the windows, saying, “You can also take away the petals that have wilted, you like to do that.” Jean replies that she does not want to water any flowers, but Pamela ignores her and goes to get the watering can in the next room. 

A little later, we bump into Jean again. She is standing by her walker in the corridor and is still looking sad. She tells Pamela that she is going to hang herself. “Why, no,” Pamela responds dismissively. Jean insists that she is going to kill herself, and Pamela gives her an absentminded pat on the cheek, saying that she could not manage without Jean, and then we continue down the corridor. 

In a later discussion with Pamela, she notes that Jean is getting worse, with more anxiety and perhaps depression. She believes it could be a great risk talking about death and dying with someone like Jean, who has suicidal thoughts. Such a conversation would only make Jean feel worse. Pamela explains that as an assistant nurse, she always has to consider the possible consequences of conversations about death and dying. The fear is also that older people with dementia may be upset with the topic. In order to manage this, any conversations about death and dying should be avoided.

## 6. Discussion

This study illustrates perceived barriers to and strategies for managing conversations about death and dying between ANs and nursing home residents. The results should be considered within a context of an overarching ideal of dying well, where health care staff are expected to help dying individuals prepare for a good and dignified death [1,13]. As the ANs were part of KUPA, an implementation project concerning palliative care [33], they also described having gained tools and knowledge for how to talk about death and dying with residents. However, the results from the 110 hours of observations of conversations between residents and ANs show that ANs used strategies to circumvent issues of death and dying raised by the residents. These issues were not understood as a need by the residents to prepare for their own death, but as a sign of illness. The results point toward the complex needs of these frail residents and the importance of multi-professional team support and supervision for the ANs to enable differentiation between healthy and unhealthy talk of death. Also, being halfway through the implementation project appears to be insufficient to support ANs in the observed conversations about death and dying with residents.

ANs need more training and supervision to ease the experienced emotional strain and communicate with different residents in a time-stressed work context. Other studies [20,22] show that nursing home staff members feel that it is difficult to find time for nonscheduled tasks such as conversations or just being by an older person’s side. Åhsberg and Carlsson [20] state that a conflict exists between what nursing assistants would like to do and what they are able to do for a dying person given the resources at hand. In our study, lack of time was perceived as an obstacle, but the overall opinion was that such difficulties must be solved, and the ANs generally seem to depend on individual strategies to find enough time.

Another barrier found in our study was the experience of emotional strain. This finding aligns with previous studies reporting that ANs find talking about existential questions to be a difficult and emotionally demanding task for which they lack competence [17,37,38]. Sundler et al. [39] also described ANs’ experience of unexpected turns in conversations when caring for older people and difficulties in detecting their needs. These situations challenged their competence, and they felt unprepared. Similar feelings of distress and powerlessness when caring for people at the end of life have been discussed by Young et al. [40]. However, the implementation project seems to have empowered the ANs with tools, to a certain degree. 

We found that the ANs used different strategies when managing conversations about death and dying. The disregarding strategy seemed to arise when the older person’s contemplations about death and dying were understood as signs of disorder, disease, and diagnosis. The AN’s reflections in this situation were concerned with health conditions of the older person and was not perceived as part of a dying process. Costello [41] calls this a curative ideology that values treatment and cures rather than death and terminal care, which, together with society’s expectations for optimal health, may contribute to how care work is performed [41,42]. Conditions such as anxiety and dementia also prompted the opinion that the risk is too great, since conversations about death and dying will intensify anxiety and perhaps increase suicidal thoughts. Therefore, a diagnosis such as anxiety, depression, or dementia, or a suspicion of this kind of condition, might be a hindrance to having conversations about death and dying. Saini et al. [43] discuss how nursing home staff attribute any symptoms to dementia and similar diseases and fail to see other possible underlying causes of symptoms and behaviors; however, multiprofessional team support and supervision are paramount in understanding these symptoms and behaviors. 

In another example, the older person is rehashing the subject of death and dying without achieving a sense of closure and readiness for the end of life. This differs from the ideal of the good dying process [7,44] grounded in palliative care and the idea of providing a good death, drawing upon a culturally established framework of how dying should occur and be experienced. Equally, in our study, when the ANs experience behavior and communication from the residents that is not in accordance with an assumed good dying process, they may respond with distraction. The notion of dying well may provide knowledge of how to proceed and what to achieve, but it also frames and decides the “right” way of dying. As Costello [41] formulates it, beliefs and ideologies about death and dying significantly shape the experiences of older dying patients and, as we have seen here, of the ANs. 

A critical question, then, is how the principles of palliative care, including existential issues, can be implemented in nursing homes [8]. According to the Advance Care Planning (ACP) recommendations from the European Association for Palliative Care [45], planning and delivering palliative care should take into account the patient’s preferences, resources, and best medical advice. However, Mignani et al. [26] point out that even when applying ACP, there seems to be a lack of communication between older people and their relatives and health care professionals about their wishes. The authors conclude that choosing the right moment and appropriate wording could have a profound impact on the value and effect of the communication, and that staff members who know the older person well are considered the ideal group to initiate an ACP discussion [26]. In Sweden, ACP is not yet standard and occurs infrequently in older people’s nursing homes. The implementation context of this study, connected to an educational intervention based on knowledge-based palliative care principles, is in line with previous findings mentioned above, and it also illustrates the complexity of these conversations that need to be addressed.

In this study, doing participant observations facilitated an understanding of what people do and how their actions change in response to different situations and contexts [46,47]. “Thick descriptions” [46] were made, where meaningful and extensive details were recorded to illustrate the element of presence and trustworthiness, thus strengthening descriptive validity [48,49]. During the observations, the first author reflected upon how her presence might affect the situations and critically reflected upon what caught her attention in each situation and why. The data analysis process was done in collaboration with the first, second, and last author, which increased the credibility.

## 7. Conclusions

This study shows that conducting conversations about death and dying can have obstacles in practice. The ANs in this study experienced increased security in conducting these conversations halfway into the seminars of a project on implementing knowledge-based palliative care (KUPA); however, barriers such as limited time and emotional stress were said to hinder the conversations from taking place. Apart from gaining tools to manage conversations, the ANs also used the strategies of distracting, comforting, and disregarding when they perceived the residents’ reflections on death and dying either as part of their illness and disease or when there was a lack of alignment between their contemplations and the ideal good dying process. The expressed ambivalence and ambiguity around conversations about death and dying should be taken into consideration in future training, with the aim of developing and supporting ANs’ communicative skills in practice. The knowledge from this study will be used and translated into guidelines for continued implementation in new nursing homes after the KUPA project is finalized. 

## 8. Data Availability

The data generated and analyzed during the current study are not publicly available due to the sensitive information with respect to a vulnerable group, older persons living in nursing homes. Therefore, before approving the study, the Regional Ethics Review Board in Lund set restrictions regarding accessibility of the data. However, after the principal researcher (GA) of the KUPA project consults with the review board, data may be available upon reasonable request.
